# Principals’ Perspectives on Joining a Kindergarten Outreach Dental Service: A Qualitative Study

**DOI:** 10.3390/ijerph191912452

**Published:** 2022-09-29

**Authors:** Hollis Haotian Chai, Sherry Shiqian Gao, Marcus Ho Tak Fung, Duangporn Duangthip, Edward Chin Man Lo, Chun-Hung Chu

**Affiliations:** 1 Faculty of Dentistry, The University of Hong Kong, Hong Kong, China; 2 Department of Stomatology, School of Medicine, Xiamen University, Xiamen 361005, China

**Keywords:** children, dental caries, dental services, oral health, qualitative research, silver diamine fluoride

## Abstract

In this qualitative study, the researchers explored principals’ perspectives on a free outreach dental service with silver diamine fluoride (SDF) therapy for children in kindergarten. Two researchers recruited kindergarten principals using purposive sampling. They conducted individual semi-structured interviews to collect the principals’ perspectives regarding their adoption of and experience with the service. They manually transcribed the interview verbatim into text and followed a thematic approach for data analysis. The researchers interviewed eight principals. The principals identified the prevalent caries status and importance of oral health promotion for kindergarten children. They acknowledged that the service enhanced dental knowledge, fostered oral hygiene practice and improved children’s oral health. To adopt this service, they needed to ensure adequate capacity to perform the service. They had no concern with staining by SDF because the parents were informed and consented to the SDF therapy. They appreciated the free service provided by a professional team managed by a reputable university. In conclusion, the principals were generally satisfied with the outreach dental service. They realised the necessity of oral health promotion. They found that parents accepted the SDF therapy although the SDF stained their children’s carious teeth. They needed support from their teachers and the children’s parents to run the service.

## 1. Introduction

The American Academy of Pediatric Dentistry defines early childhood caries (ECC) as the presence of one or more decayed, missing or restored teeth in the primary dentition of a child younger than 71 months [1]. ECC is the most common chronic childhood disease affecting a considerable number of preschool children worldwide [2]. A systematic review describing the prevalence of ECC among 5-year-old children globally showed that most of the identified publications reported an ECC prevalence of more than 50% [3]. In Hong Kong, approximately half of all 5-year-old children suffer from ECC, and 90% of these caries are left untreated [4]. ECC can cause pain and infection and advanced ECC eventually progresses into the tooth pulp to form a dental abscess [5]. Untreated carious teeth can lead to tooth loss and compromised dentition. More importantly, compromised dentition significantly affects the child’s nutrition as well as their growth, development and general health [6]. The performance of therapeutic procedures in young children is still a great challenge for dentists, especially when they treat child patients who are very young and uncooperative [7]. Therefore, the application of minimally invasive approaches is of the utmost importance. Silver diamine fluoride (SDF) treatment has been proposed for this purpose as a non-invasive, simple, quick, painless and effective method in managing ECC among young children [8]. A systematic review showed that 38% SDF has a statistically significant caries-arresting effect on primary teeth, with 81% of treated ECC becoming arrested [9]. Apart from staining the arrested lesion, no significant complications were reported [10].

The Faculty of Dentistry of the University of Hong Kong has been providing kindergarten-based outreach dental services to serve kindergarten children in Hong Kong since 2008 [11]. This outreach dental service involves three components: oral health examination with SDF treatment, oral health promotion for parents, and teacher training. The parents’ and kindergartens’ acceptance of this service program is very high. The parents reported no significant complications during these years. The kindergartens were also satisfied with this programme, and 97% of them considered this service helpful for promoting oral health in young children. However, no detailed perception or suggestions from the principals have been obtained. In addition, deciding to adopt an oral health promotion service with treatment and research components can be more complicated than adopting other programmes that involve oral health education or tooth brushing. Therefore, a qualitative study was conducted, the aim of which was to explore the principals’ perspectives towards the adoption and their experience of the kindergarten outreach dental service.

## 2. Methods

### 2.1. Study Design

A qualitative approach was used to collect the principals’ perspectives regarding their adoption of and experience with the kindergarten outreach service. A grounded theory underpinned the research. The ethics approval was granted from The Institutional Review Board of the University of Hong Kong/Hospital Authority Hong Kong West Cluster (Approval No: UW 16-518). Principals from kindergartens that joined or rejected the outreach dental service were invited to participate in this study. Semi-structured interviews were employed as the data collection method, and thematic analysis was used for data analysis. The reporting of this qualitative study follows the Consolidated Criteria for Reporting Qualitative Research (COREQ) checklist.

### 2.2. Sampling

Ten principals who had adopted this outreach dental service were purposefully selected based on districts and funding types. Invitation letters were sent to them in which the study’s purpose and procedures were explained. The principals were encouraged to ask questions related to the study to facilitate informed decision making. All selected principals needed to be able to communicate in Chinese (Cantonese) because the interviews were conducted in Cantonese. No further criteria were set for the recruitment. Ultimately, eight of them completed the semi-structured interview before data saturation was achieved.

### 2.3. Data Collection

The researchers were especially interested in the participants’ experiences and perceptions of the outreach dental service. Based on the researchers’ clinical and research experiences and the issues discussed in the literature, an interview guide was developed to provide a practical framework. Topics for interviews with the participants included (1) adoption and (2) experience. The adoption part focused on the kindergartens’ experience of health promotion programmes as well as the factors and concerns before they adopted the outreach dental service. The experience part explored issues during the implementation period as well as the principals’ satisfaction with and suggestions regarding the whole outreach dental service. The interview guide was passed through the research panel and pilot tested on laypersons to enhance the study’s rigour. Revisions, including the use of exploratory questions and the questions’ wording, were made until the research team reached agreement. Table 1 presents the main and follow-up questions of the two topics in the semi-structured interview.

Two trained PhDs (one female and one male) and one trained master’s student (female), all of whom were public health researchers, conducted the study. They did not know the participants personally before the study. Each interview was held in a room of the principal’s kindergarten. Participation was voluntary, and the participants could choose to stop at any time without negative consequences. A research assistant recorded field notes throughout the interviews. The interviews lasted approximately 60 min. All the interviews were audio-recorded and then transcribed manually. All transcripts were checked against the original recordings for accuracy. All personal information and the interviews’ contents were kept confidential and only used for research purposes. No repeat interviews were carried out, and the transcripts were not returned to the participants for comments or corrections because the audio recordings were collected to guarantee data accuracy.

### 2.4. Data Analysis

The audio-recorded data were transcribed verbatim and checked against the original recordings. The field notes were added to the transcription, if indicated. A thematic analysis approach was adopted by the facilitators and research assistant. An initial thematic framework was constructed by listing, reducing and grouping previously detected topics into a set of themes and subthemes. The initial thematic framework was refined and finalized to a final thematic framework based on the detected patterns among the data. The data was further explored based on the refined thematic framework. The obtained data was then displayed in a set of matrices. Each theme had one matrix where the researchers started to add their comments and more analytical views on the matrices. Lastly, the researchers summarized the data obtained according to themes and identified possible linkages among the themes for further reporting of the information.

## 3. Results

The principals’ perspectives were categorised into two main themes: (1) factors and concerns regarding the service adoption and (2) experience of the service.

### 3.1. Factors and Concerns Regarding the Service Adoption

**Figure 1:**
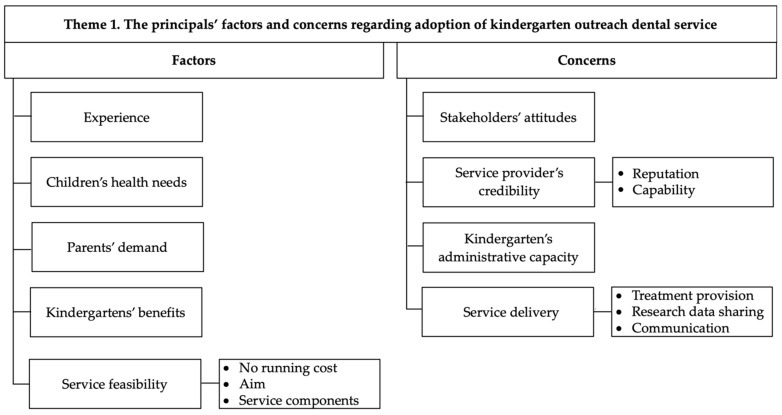
The principals’ factors and concerns regarding adoption of the kindergarten outreach dental service.

Figure 1 shows the principals’ factors and concerns regarding adoption of the kindergarten outreach dental service.

#### 3.1.1. Factors of the Service Adoption

The service adoption discussion could be grouped into five aspects: experience, children’s health needs, parents’ demand, kindergartens’ benefits and service feasibility (Figure 1).

Experience—Most of the principals had experience in adopting kindergarten activities for health promotion. Some principals also had experience in organising health promotion activities. The common health promotion activities were spine protection, foot examination, eye examination and dental clinic visits.

Children’s health needs—Most of the principals were able to identify the treatment needs of common diseases among kindergarten children. Regarding oral health problems, the principals mentioned signs of the pupils’ badly decayed teeth. They also noticed the students’ lack of concentration and inability to eat well because of painful teeth. Beside dental problems in one kindergarten, some principals also mentioned the high caries prevalence among Hong Kong preschool children, indicating that they have a high awareness of preschool children’s oral health needs. All the principals mentioned that they believed this service could improve their students’ oral health. In addition, oral health knowledge and awareness could also be enhanced. Their pupils could obtain information about their oral health status after the dental examination, i.e., knowing that they had dental caries. Moreover, they would care more about their teeth and be motivated to maintain good oral health conditions, which in turn could foster their oral hygiene practice.

*‘We used to have a child with a dental abscess. His face was swollen, and the teeth were very painful. In fact, his mental state was already very poor, and he had no energy in class. I think the kid’s motivation of learning had been affected on those days.’*

—Principal No. 2

Parents’ demand—Principals mentioned some parents have a low awareness of the importance of good oral health and don’t pay attention to their children’s oral health status, which increases the necessity of oral health promotion programmes to improve parents’ dental knowledge. However, many parents also reported to the kindergartens that even though they wanted dental examinations for their children, the cost was high in Hong Kong. In addition, the general dental practitioners might refuse to treat young children; therefore, parents encountered difficulty in finding paediatric dentists. Because of the low accessibility of regular dental check-ups and low availability of dentist information, the principals were willing to adopt this kind of outreach dental service. Meanwhile, the parents also showed their trust by allowing the kindergartens to choose health promotion programmes benefitting their children. Principals believed that parents’ dental knowledge and awareness of their children’s oral health status could be enhanced after they joined the programme. Children’s oral hygiene practices also could be improved. For example, parents might start helping their children with tooth brushing and rinsing their mouth after meals. They might also start to modify their children’s diets to establish a better oral health status for their children.

*‘There are many parents who don’t know the importance of brushing their children’s teeth. We have attended several “Love Teeth” programmes for several years, but they have not helped much. Second, I was thinking about why the children now have more chances of tooth decay. It’s not because the parents don’t know how to help their children; it is because of the high cost of dental visits and the school dental care service serves children in primary school but not in kindergarten.’*

—Principal No. 3

Kindergartens’ benefits—A health-promoting school is a place where all school community members work together to provide students with integrated and positive experiences and structures that promote and protect their health. Although the principals did not mention the term ‘health-promoting school’, they mentioned the key concepts of a health-promoting school. Most of the participants were able to recognize and point out the importance of good health for good learning. On the practical side, although it is not mandatory for the principals to develop health programmes for the students, some principals understood the importance of good health and were internally motivated to seek programmes that could promote their students’ health. Some principals also mentioned the possibility of incorporating the content of the health-promoting services into the school curriculum to facilitate teaching because they realised there was a lack of dental knowledge in the curriculum and that teachers and students had a relatively low awareness of good oral health. The principals believed that the programme could enrich the health-related activities in their kindergartens. With this programme, they could also improve their kindergarten’s reputation. Some principals also mentioned the expectation of generating more positive attitudes towards health-related practices in the wider community.

Service’s feasibility: No running cost—Principals stated that they would avoid services organised by profit-making organisations that involve a service charge or selling of products after the service. Adopting a free-of-charge service could avoid creating barriers for students who were not able to afford the service. The free service creates less complicated administrative work for the school and would not create the impression that the school is earning money from parents.

Service’s feasibility: Aim—Principals preferred services with a clear and direct aim. They would determine whether the proposed service’s aim addressed the kindergarten’s concern. Services intended to aid the grassroots population were also popular among kindergartens. For topics that were too general, the school might not use additional resources to adopt a new service. Moreover, services that could benefit more students would receive priority. For services addressing rare diseases, the school might not put in additional resources.

Service’s feasibility: Service components—The principals preferred programmes with structured content. All the principals agreed that the outreach dental service’s three components had been clearly stated during the introduction period. For the ‘dental outreach service’ component, the principals appreciated that the dental examination would be provided because preschool children in Hong Kong had no organised dental care. They preferred the service to be conducted in a low-risk environment with simple and clear setting requirements. For the ‘oral health promotion for parents’ component, the oral health talk and individual consulting provided would establish a direct communication with parents, which the principals welcomed. Regarding the ‘teacher training’ component, the principals hoped that the knowledge and skills learned could be integrated into kindergartens’ thematic curriculum.

#### 3.1.2. Concerns about the Service Adoption

The principals’ concerns could be grouped into four main aspects: stakeholders’ attitudes, service provider’s creditability, kindergarten’s administrative capacity and service delivery (Figure 1).

Stakeholders’ attitudes—The principals stated that the dental service’s outreach mode would be more acceptable to their children if the providers were familiar with the kindergarten environment as well as the teachers and classmates. Some principals also mentioned that the children might feel the dental examination was enjoyable if the teachers would explain the service in an interesting way. Students also showed a more positive attitude towards the dental examination if they received stickers or other presents when they finished the examination. If a suitable service could be identified, the principals would also explore the teachers’ and the school board’s views. They would try to determine whether the teachers were reluctant to squeeze in extra time and increase their workload to help with the service’s implementation. If the teachers also agreed with the service’s potential benefits and were willing to help, the possibility of adoption would increase. Some principals mentioned that the school board showed a positive attitude towards the service and supported the adoption decision.

*‘Usually, when we have a new plan, we will share information with the teachers, such as what the service is and why we want to participate. In fact, we will ask for the teachers’ opinions. In addition, we have a Parent-Teacher Association. We will conduct a survey within the association first when necessary.’*

—Principal No. 7

Service provider’s credibility: Reputation—For the service provided, the principals preferred to select services provided by organisations with public credibility and an effective regulatory system. They believed these well-recognised organisations could provide good support and take responsibility for their services, which was difficult for private providers. The principals would consider the services’ continuity by checking the organisations’ relevant track record in the specific kind of health-promotion experience. The level of trust would be higher if the organisation had verifiable experience in benefitting a large number of participants and could provide more resources in their professional area. Principals believed an organised organisation could ensure service quality by providing its staff with quality training. Parents also often accepted such reputable organisations. Some principals stated that they did not have to do extra research regarding the service content because of the organisation’s trustworthiness due to their background and professionalism.

*‘I’ll check whether the background of the service provider was from those large-scale research institutes or universities that are able to provide a good support. It’s not good if we accept a service that may suddenly be stopped in the middle of implementation. We want to see the positive effect on our students after the completion of the whole programme.’*

—Principal No. 1

Service provider’s credibility: Capability—Principals would also consider the service providers’ capabilities. They believed a more extensive educational background could enhance its professionalism. Meanwhile, service providers who had experience in attending or organising other similar health promotion programmes were more trustworthy.

Kindergarten’s administrative capacity—All kindergartens in Hong Kong are privately run and can be categorised as non-profit kindergartens and private independent kindergartens, depending on their sponsoring organisations, which can be either voluntary agencies or private enterprises. Most kindergartens operate on a half-day basis, but some offer whole-day classes. Principals mentioned that the kindergarten’s nature would influence their consideration of the programmes. Kindergartens vary greatly in their scale of operation, and the number of classrooms can range from two to over ten. Principals would decide whether to adopt the service based on the kindergarten’s administrative capacity.

Service delivery: Treatment provision—The principals would consider whether the programme’s content was suitable to provide in a kindergarten setting. Those programmes may cause too much burden on students who would be rejected. In terms of the SDF treatment provided in the programme, some principals showed concern about SDF’s effectiveness and the black staining. They wondered whether the caries-arresting effect was stable and what would be the level of blackness. They were also afraid the black staining on the anterior teeth would influence their pupils’ appearance for a long time. Some principals revealed that black staining on circumjacent skin might also be a problem. All principals emphasized the importance of explaining the SDF treatment’s effectiveness and potential adverse effects to kindergartens, parents and students. Then, the kindergartens and parents could conduct a trade-off analysis between the benefits and the black staining. Parents worried about their children’s appearance could have the choice to accept only the dental examination without SDF treatment when filling out the consent form. Most of the principals believed parents would show a high level of trust in the service provider’s professionalism.

Some principals did not show many concerns about the black staining because they knew staining on the posterior teeth would not be a problem and that the primary teeth would exfoliate. Principals would not select treatments that included traumatic procedures, particularly those involving injection or tooth drilling. They found these procedures unsuitable to be carried out in a school-based setting because of their traumatic nature. These activities should be provided in a healthcare setting in the presence of parents. The principals could not predict the children’s response. They worried about the children having some hidden diseases that might be triggered during the stressful process. They were also concerned that some students might be too frightened and cry, which would make the situation uncontrollable.

*‘I don’t have such big worries in terms of the SDF treatment because I understand that SDF is really helpful for children in preventing tooth decay. There were parents who worried about the black staining during the process. At that time, we called the dentist to communicate the worries. Parents’ doubts were dispelled after the clear and instant explanation by the dentist.’*

—Principal No. 1

Service delivery: Research data sharing—For services that involve research components, the principals were open-minded and knew the importance of research in advancing knowledge and services. However, they would only adopt a service that would share the research findings. The results should be shared to facilitate the school’s development and to improve the health of students. Moreover, they would refuse programmes that treated students simply as study subjects and did not provide a service.

Service delivery: Communication—All the principals agreed that the communication with the service team was important. Clear service content should be provided, particularly regarding adverse effects that may occur. An introduction could facilitate their understanding of the service. The principals preferred having one person in charge, whom the school could contact to increase communication efficiency. During the service, most principals stated the language barriers would not cause a problem because they were more concerned about the service team’s professionalism. However, some principals preferred local dentists because they were afraid the language barrier would reduce the children’s sense of security. In the case of an emergency or if parents wanted to ask questions related to the service, a phone number should be provided for contact. The service team should also be able to provide a follow-up service in case of any adverse effects or queries.

### 3.2. Experience of the Service

**Figure 2:**
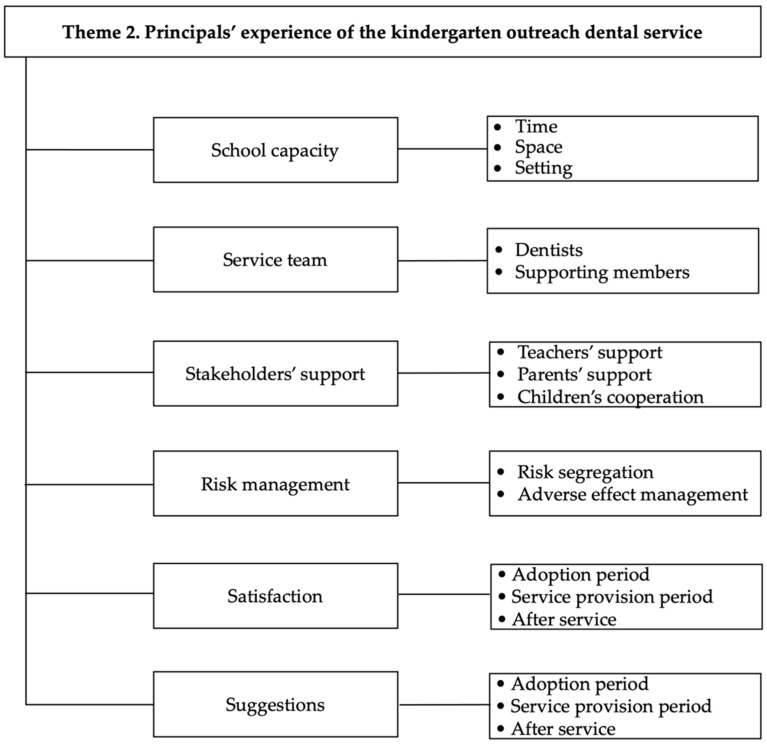
Principals’ experience of the kindergarten outreach dental service.

The discussion about the experience could be grouped into six aspects: school capacity, service team, stakeholders’ support, risk management, satisfaction and suggestions. Figure 2 shows the principals’ experience of the kindergarten outreach dental service.

School capacity: Time—Some principals were concerned about scheduling the service date. They preferred to plan the service date two to three months in advance to adjust their school schedule for other curricula and activities. They hoped the service team could provide an estimate of how long the whole service would take, especially for half-day kindergartens. Some students may have personal schedules that conflict with the service. Some principals mentioned they might divide the students into small groups to ensure the programme’s smooth operation.

School capacity: Space and setting—Some principals stated that their buildings were spacious and the setting for the dental examination would not cause a problem for them. Other principals were concerned about the setting because their available space was relatively limited. They would need to remove or relocate many items to fulfil the requirements. Principals also mentioned that they were very familiar with the setting.

*‘How to lay out the tables, whether pillows and towels are needed, etc. All this stuff needs to be improved gradually. We were confused for the first time and got more and more familiar later.’*

—Principal No. 3

*‘The biggest problem is the space problem. If the space is not big enough, we have to empty the places originally for physical fitness, music and various other activities to make more room for the programme.’*

—Principal No. 5

School capacity: Workload—All the principals stated that although the workload during the preparation period was heavy, they were willing to use this time for their pupils. After they became familiar with the service, it would be smoother and easier for them to conduct the programme.

Service team: Dentist—The principals appreciated the dentists’ careful operation, especially during the treatment provision. They found the communication between dentists and children important. The dentists’ eye contact, tone, intonation and gestures can influence the children’s performance. The dentists became more familiar with the students, increasing the children’s sense of security.

Service team: Supporting members—The principals mentioned that sometimes the responsible contact person was not part of the several teams that arrived on campus, which made communication more difficult.

Stakeholders’ support: Teachers’ support—The principals stated that health professionals could carry out the examination and treatment more easily with the teachers’ support. Most of the teachers were willing to help even though their workload increased. Before the service, the teachers needed to distribute and collect consent forms from parents. On the service day, the teachers would introduce the service to students in advance and encourage them to participate. Some principals also asked teachers to be present throughout the process to ensure their students’ safety and the smooth operation of the programme. If an accident occurred, the whole process could be restarted if the teachers were present. After the service, suggestions from participating teachers would be collected. They would try to simplify the work to reduce the teachers’ workload. Some principals also mentioned that if the teachers were already overloaded or too many complicated tasks were assigned, the teachers might not be able to help.

*‘I feel that the service has so many benefits for the children. We just arrange time for implementation. There might be a lot of administrative work at an early stage, such as scheduling the service and sorting the materials. Although it takes more time, we can still afford it and are willing to provide this opportunity for dental examination to our children.’*

—Principal No. 2

Stakeholders’ support: Parents’ support—The principals all agreed that the service could not be conducted smoothly without parents’ support. The parents were required to sign the consent form before the programme. They were also encouraged to attend the oral health talks and participate in individual counselling if needed. Some parents had little idea about their children’s oral health status. Some had concerns about the service (i.e., black staining of SDF treatment). The principals insisted that the parents should receive sufficient information to realise the importance of good oral health, which could increase their acceptance of the programme.

Stakeholders’ support: Children’s cooperation—Some principals mentioned that the student’s cooperation was also essential for the programme. Children’s dental fear may come from bad experiences or from the setting. The principals said they would try to help these children in collaboration with parents and teachers to reduce their fear and improve their cooperation with the programme.

Risk management: Risk segregation—All principals emphasized that the organisation should provide parents with a consent form. The parents who sign the consent form should indicate their understanding of the service and that it is voluntary participation. This form would allow the school to bear less liability for the treatment provided and ensure that the parents understood that it is the service provider rather than the school who bore responsibility for the treatment. The principals also said that students who had diseases with a stigmatising effect would be treated more carefully.

*‘The consent form can be regarded as a risk segregation from kindergarten to parents.’*

—Principal No. 4

Risk management: Adverse effect management—The principals mentioned that it was vital for the organiser to provide clear service content, particularly regarding possible adverse effects. In the case of an emergency or queries related to the treatment, a contact phone number was a necessity. The arrangement of a follow-up or referral for suitable further treatment was also important.

*‘If the service organiser can provide sufficient information for parents during the planning period and let them know what the follow-up is like after those treatment activities as well as what are the benefits for the children, I think these are the important factors I will consider.’*

—Principal No. 2

Satisfaction—All the principals were generally satisfied with the service adopted. They considered the service provider a professional team and confirmed the benefits their pupils received.

Satisfaction: Adoption period—During the contact period before adoption, the principals mentioned that the organisation had already provided sufficient materials to introduce the programme’s details. The kindergarten did not have to prepare extra materials for the parents. The communication between the kindergarten and service provider was efficient. The consent form was also clear and allowed parents to accept only a dental examination without the treatment. The principals also mentioned that the service team’s replies to inquires was always timely.

Satisfaction: Service provision period—All the principals were satisfied with the service provision procedures. They stated that the whole service team could maintain high work efficiency even though the students’ order did not strictly follow the name list. The handling of uncooperative students was also appropriate without the need for physical restraint. Principals also mentioned that the service provider would try to reschedule the service for absent students, which showed their responsible attitude towards the programme. They believed that the kindergarten could handle several teams in one service as well because they were familiar with the procedures already.

Satisfaction: After service—Principals were satisfied with the reports the service team provided. They stated the report was simple and clear to understand. The principals noticed an improvement in their pupils’ oral health status. The students also showed a supportive attitude towards the programme. Some principals mentioned their pupils paid more attention to their teeth after the service and were motivated to maintain good oral health conditions. The SDF treatment was acceptable and did not affect the students’ daily lives. The principals mentioned that most of the parents had positive comments about the programme. Only a few parents raised concerns about the black staining, but those were resolved after talking with dentists. The parents’ awareness of their children’s oral health status and dental knowledge improved. They started to assist their children with tooth brushing and cared more about their children’s diet, which in turn increased their confidence in the whole programme. Principals also stated the successful launch of the service enhanced their school’s image among parents and the school management committee.

Suggestions: Adoption period—The principals suggested confirming the service date as early as possible and trying not to change the scheduled time frequently because the kindergartens need time to plan for school activities. It would be better if the service team could provide pictures or videos to show the setting and procedures to ensure smooth operation on the service day. Principals also suggested the service team should check in advance to see if anything was missing or if there was a change in the materials. Otherwise, it might create extra workload for the kindergarten to prepare new materials or adjust the setting on the service day. All the principals mentioned the importance of efficient communication. They preferred one responsible person to handle communication. They should also be informed about any important change in team members (i.e., the dentist) in advance. For the oral heath talk and teacher training, the principals suggested that an outline of the content be provided so the parents and teachers could prepare questions to ask in advance.

Suggestions: Service provision period—Some principals suggested that the dentist could have a brief communication with the students before the service to calm them down. They could demonstrate what happens by using age-appropriate terms, and children might be allowed to touch or handle the equipment. During the service, the dentists’ skills in handling uncooperative students could be enhanced. It would be desirable that they pay more attention to the children’s emotions and to speak to them in a supportive manner.

Suggestions: After service—The principals suggested more referral or follow-up information be provided for severe cases after the clinical examination.

### 3.3. Reasons for Rejection

The researchers contacted kindergartens that rejected the outreach dental programme, inviting them to participate in the qualitative study. Most of them declined the interview meant to explore their concerns regarding joining the outreach dental programme.

Based on the information collected during the service invitation period, the common reasons for kindergartens failing to join the outreach dental service included the effects of COVID-19 (33%), kindergartens’ administration issues (19%), lack of interest (10%) and similar projects’ progress (3%). In addition, of the kindergartens contacted, 35% did not respond to our invitation. A considerable proportion of the principals were concerned about the unstable COVID-19 situation. Their focus would be the difficulties in shifting work arrangements and obtaining consent from the stakeholders. Some principals also considered that their kindergarten was unsuitable to attend the outreach dental services because of various administration issues, such as limited space, a tight schedule, limited personnel, low participation rate, outsider onsite-service prohibition, instructions from organizations and religious issues. Some kindergartens rejected the program simply due to a lack of interest. Several kindergartens mentioned they had joined a similar oral health promotion project or had their own dental service provided by family, schools or an organisation.

## 4. Discussion

This qualitative study explored principals’ perspectives on and their experience of adopting an outreach kindergarten dental service through semi-structured interviews. Data gathered have provided public health professionals with information on the recipients’ views of the oral health promotion service in their natural context. The results provided implications for strategic planning of more school-based health promotion programmes. The findings also enabled public health professionals to improve medical communication with community parties, thereby promoting the continuous improvement of care delivered to the community.

The qualitative research method was adopted in this study because it allowed for the systematic collection, organisation and interpretation of textual material obtained from the principals [12] and allowed for the exploration of their experience in their natural context [13]. The semi-structured interview, the most frequently used data collection method in the healthcare setting, allows the research topic to be explored from participants’ perspectives in depth and in detail [14]. Thematic analysis was employed as the data analysis approach in this study based on the research question’s descriptive nature. This approach can help researchers to identify and analyse patterns of meaning in the collected data by generating codes and deriving a thematic framework [15].

The decision of whether to adopt a new health promotion service is not an easy one for an educational organisation, particularly when the treatment and research are conducted in the school environment. Although studies have been conducted on the factors that address school managers’ concerns, those studies have been based on school managers’ views at the formative stage rather than at a school that has already completed a health promotion service [16,17]. The kindergarten needs to consider many factors and address concerns before deciding to join the programme. However, limited studies had been conducted on the factors that affect that decision.

In this study, the factors that influenced the adoption of an outreach dental service were examined. Before adopting an oral health promotion programme, the principals had considered many factors and ensured that the service could be safely and effectively carried out. Their adoption of a new health promotion service involved identifying the students’ health needs and was initially intended to improve the students’ oral health. Then, strategic planning of feasible programmes would be conducted, including considerations about the service structure, the service provider’s trustworthiness, administrative capacity and stakeholders’ views. Regarding the major concern of the SDF treatment staining students’ teeth black [18], a clear explanation before the treatment and an effective complaint-handling process are important to increase acceptance. This finding was consistent with previous research evaluating parents’ acceptance of the use of SDF [19].

The study also showed that stakeholders’ support is crucial for the whole programme’s smooth operation. Therefore, future planners of an oral health promotion service may want to address stakeholders’ requirements and increase their awareness. For instance, programmes could coach parents on helping their children maintain good oral health and empower teachers to deliver effective oral health messages to children in kindergartens. Interventions and programming for parents could be facilitated at their child’s school, thereby generating a shared commitment to work together with the school staff and teachers [16]. No matter which oral health promotion programmes have been adopted, practical problems can arise at every stage of implementation [20]. Careful considerations based on local situations and proper solutions need to be addressed between the service team and the school.

According to the Ottawa Charter, health promotion is the process of enabling individuals and communities to increase control over the determinants of health, thereby improving health for an individual to live an active and a productive life [21]. The principals also mentioned health promotion needs to strengthen community action and reorient health services based on preventive strategies. Underpinned by the values set out in the Ottawa Charter, there is increased attention on the school as a health-promoting setting [22]. The influence of childhood experience on health status later in life has put kindergartens in a central position to deliver effective interventions.

The health-promoting school model includes changes in three points: (a) formal health curriculum, (b) the school’s ethos and environment and (c) engagement with families, communities or both [23]. Although health promotion is not a prime focus for schools, some principals understand its importance and are willing to adopt a number of health promotion programmes in certain situations. Taking into account the principals’ perspectives is important because these views help form a more complete picture of how school managers work with health promotion and what is needed to enhance health promotion to improve students’ opportunities for learning and a good life. The findings can provide meaningful implications for health professionals in the planning of more school-based health promotion programmes that fit the school’s concept.

The results also included the rejection reasons for the outreach dental programme. Some kindergartens were hesitant to adopt an outreach dental service during the COVID-19 outbreak. However, health professionals should realize that COVID-19 has weakened the provision of public dental care, especially for children who have experienced serious dental disease. Meanwhile, the need has increased for prevention and access [24]. Therefore, opportunities to promote preventive dental practice should be taken, and more effort should be made to communicate with the public, including messages about the importance of promoting and maintaining good oral health [25]. Some kindergartens showed low interest in an outreach dental programme. It is also vital to organise, maintain and explore ways to collaborate among various stakeholders to allow knowledge transfer to benefit a wider community [26].

This study had some limitations. First, no principals from kindergartens that rejected the outreach dental service agreed to attend the interview, resulting in a lack of detailed information regarding their reasons for rejection. Second, the interviews and coding were conducted in Chinese, but the themes for analysis were generated in English. Twinn (1998) studied the effects of translation at the transcription stage versus using the original language for transcription and then translating the codes and themes into English. Studies have highlighted the importance of analysing data in the language of the interview rather than translated data to avoid compromising the quality of data obtained from non-English speaking populations [26,27]. Therefore, a backward translation of the themes into Chinese was conducted to check the data’s reliability. The researchers gained in-depth information about the principals’ experiences with and perspectives on the outreach kindergarten service. These findings provided insights into strategic planning of school-based health promotion programmes.

## 5. Conclusions

This qualitative study showed that the principals were generally satisfied with the kindergarten-based outreach dental service with SDF therapy. The principals were aware of the necessity and importance of oral health promotion in kindergarten. They found parents accepted SDF therapy although SDF stained their children’s carious teeth. They particularly welcomed this service because a university provided it, and it was free of charge. However, they needed support from the teachers and the pupils’ parents before they adopted the programme.

## Figures and Tables

**Table 1:** Main and follow-up questions of the two topics in the semi-structured interview.

Main Questions	Follow-Up Questions
Topic 1. Adoption	
Experience of school-based health promotion programme	How was your experience of the school-based health promotion programme? How was your experience of the school-based oral health promotion programme?
Adoption of the outreach dental service in kindergarten	What is your view of your students’ oral health conditions? What are possible effects of the outreach dental service? What factors would cause you to adopt the outreach dental service? What factors can prioritize this outreach dental service over other activities?
Concerns before adopting the outreach dental service in kindergarten	What concerns do you have before adopting the outreach dental service? What would make you consider the provider reliable and capable? How do you think research is incorporated in the outreach dental service? Why would you accept the caries-staining SDF therapy in this service?
Topic 2. Experience	
Implementation of the outreach dental service in kindergarten	What are the factors that ensured the smooth operation of the service? What support do you need to ensure the safe execution of the service? What are the difficulties during the implementation of the service? Under what circumstances would you stop the service when it is implemented?
Satisfaction with and suggestions regarding the outreach dental service	How satisfied were you with the service? What suggestions do you have to improve the service?

## Data Availability

The data sets generated and/or analysed during the current study are available from the corresponding author upon reasonable request.
